# Rotational stability and refractive outcomes of the DFT/DATx15 toric, extended depth of focus intraocular lens

**DOI:** 10.1007/s10792-023-02673-7

**Published:** 2023-03-10

**Authors:** Kevin M. Barber, Sara O’Connor, Philip Mackinder, Andreea Chih, Brian Jones

**Affiliations:** Central Florida Eye Specialists, 968 International Parkway, Lake Mary, FL 32746 USA

**Keywords:** Vivity, Extended depth of vision, Intermediate visual acuity, Corneal astigmatism correction

## Abstract

**Purpose:**

To quantitatively assess postoperative rotational stability and visual acuity with the DFT/DATx15 extended depth of focus (EDOF) toric intraocular lens (IOL).

**Methods:**

In this prospective case series, thirty-five patients with a calculated IOL power between + 15.0 D and + 25.0 D, corneal astigmatism between 0.75 D and 2.25 D, and no significant ocular pathology underwent cataract surgery. Primary outcome was rotational stability of the IOL at 1 month post-operatively. Secondary outcomes included residual refractive astigmatism, absolute residual astigmatism prediction error, and monocular distance and intermediate visual acuities.

**Results:**

Mean absolute postoperative IOL rotation was 1.1 ± 0.2 degrees, with no rotation of more than 3 degrees at the final visit. Monocular mean best spectacle-corrected distance visual acuity (BSCDVA) improved from logMAR 0.27 ± 0.030 to 0.078 ± 0.017 (*P* < .001). Monocular uncorrected distance visual acuity (UCDVA) improved from 0.93 ± 0.096 to 0.18 ± 0.022 (*P* < .001). Best spectacle-corrected intermediate visual acuity (DSCIVA) was 0.17 ± 0.025, and uncorrected intermediate visual acuity (UCIVA) was 0.27 ± 0.040. Residual regular astigmatic refractive error was 0.21 ± 0.047 D.

**Conclusions:**

The toric DFT/DATx15 EDOF lens showed excellent rotational stability and effective and predictable correction of astigmatism. Its refractive outcomes and safety profile were similar to those identified in prior studies of the non-toric DFT/DAT015 EDOF IOL. A small difference in monocular BSCDVA, of uncertain clinical significance, was found when comparing these outcomes with prior DFT/DAT015 data. The trial was retrospectively registered on November 5, 2021 (TRN ​​NCT05119127).

## Abstract (plain language summary)

In cataract surgery, the natural lens of the eye is replaced with an artificial lens implant. In many cases, the patient’s glasses prescription in the operated eye can be reduced or eliminated through careful choice of a lens implant. There are many types of lens implants available. Toric lens implants are used to reduce one component of the glasses prescription, called regular astigmatism (or often just “astigmatism”). To maintain the full astigmatism-reducing effect of the toric lens, the lens implant must not rotate significantly within the eye after the surgery. The DFT/DATx15 (Vivity™) is a relatively new type of lens implant designed to offer patients good spectacle-free vision at far distances and improved glasses-free vision at arm’s length (“intermediate”) compared to a more traditional lens implant that is designed to maximize spectacle-free distance vision only. This study reports one surgeon’s experience with measuring the amount of rotation of DFT/DATx15 lenses after surgery. This study also assessed the ability of the DFT/DATx15 to reduce regular astigmatism and improve glasses-free vision at far and intermediate distances. The results show that this lens did not rotate significantly within the eye and was effective at reducing the regular astigmatism as intended.

## Introduction

Patients with clinically significant regular astigmatism typically require spectacle correction to achieve optimal visual acuity. Regular astigmatism can often be reduced or eliminated at the time of cataract surgery by using a toric intraocular lens (IOL) to compensate for corneal astigmatism. Successful use of a toric IOL requires maintaining precise alignment of the marked flat meridian of the IOL with the steep meridian of the patient’s corneal astigmatism. Postoperative rotational stability of the IOL is therefore of great interest to the cataract surgeon. The efficacy of toric IOLs in correcting corneal astigmatism has been demonstrated in numerous studies; a 2016 meta-analysis [1] of 13 randomized controlled trials comparing toric vs. non-toric IOLs found that use of a toric IOL was associated with higher postoperative uncorrected visual acuity and a higher fraction of patients reporting postoperative spectacle independence for distance vision. Rates of repositioning surgery have been found to be low [2–5]. The AcrySof® toric IOL has been found to be associated with a lower degree of rotation than the TECNIS® toric IOL in some [4, 6–9] but not all [10] studies.

Numerous approaches are available for improving or preserving near and intermediate vision after cataract surgery, [11–15] each with its own advantages and trade-offs. These include spectacles and contact lenses, monovision, multifocal and enhanced depth of focus IOLs, pinhole IOLs, pharmacologic miosis, and corneal inlays. The DFT/DATx15 (Acrysof Vivity™; Alcon, Fort Worth, Texas, USA) intraocular lens is a single-piece soft hydrophobic acrylic lens featuring a proprietary non-diffractive anterior surface geometry [16]. It is designed to yield improved uncorrected visual acuity at intermediate distances, as compared to a traditional monofocal design, without sacrificing uncorrected distance visual acuity and while minimizing visual artifacts and loss of contrast sensitivity. Premarketing approval (PMA) data submitted to the FDA for the non-toric model (DFT/DAT015) of this lens demonstrated [17, 18] superiority of monocular distance-corrected intermediate visual acuity and noninferiority of best-corrected distance visual acuity to within 0.1 logMAR units, under photopic conditions, compared with a monofocal IOL. A recent study [19] under real-world conditions confirmed these results.

The DFT/DATx15 lens also is available with a toric posterior surface similar to that of other Alcon toric IOLs, such as the SN6AT series, whose safety, efficacy, and rotational stability have been previously evaluated [20–29]. Because of this physical similarity, clinical testing specifically of the toric model of the DFT/DATx15 lens was not required for FDA approval, and data regarding its rotational stability have not previously been available.

This study was therefore undertaken to assess the refractive performance of the toric models of the DFT/DATx15 EDOF IOL, with particular attention to rotational stability. To our knowledge, this report provides some of the first real-world data regarding rotational stability and visual outcomes in patients with regular corneal astigmatism implanted with this type of lens.

## Materials and methods

### Study design

Patients were recruited between September 30, 2020, and February 28, 2021. Informed consent was obtained from all patients. This study conformed to the principles of the Declaration of Helsinki, ISO 14155:2011, and all other applicable regulations, and was approved and monitored by the Institutional Review Board as protocol number 63171943. This trial was registered at www.clinicaltrials.gov with registration number TRN ​​NCT05119127. Individual deidentified patient data will not be externally shared. The following description includes information from the unpublished study protocol.

Inclusion criteria were as follows: eligible subjects were those at least 45 years of age undergoing cataract surgery with intraocular lens implantation who elected placement of a DFTx15 or DATx15 toric EDOF IOL. Only eyes requiring a calculated IOL power between + 15.0 D to + 25.0 D and having regular corneal astigmatism correctable with one of the study lenses (corresponding to keratometric astigmatism values of approximately 0.75–2.25 D) were included. Finally, subjects had to be willing and able to adhere to all scheduled visits and undergo all other study procedures. For patients with two eligible eyes, only the first eye to undergo cataract surgery was included in the study.

Subjects were required to have no other identifiable ocular pathology potentially compromising visual acuity. Only subjects with a postoperative visual potential of 0.2 logMAR (Snellen equivalent 20/32) or better in both eyes, in the opinion of the investigators, were considered eligible. Other exclusion criteria included clinically significant corneal dystrophies or a history of corneal refractive surgery, abnormalities of the pupil, uveitis (whether infectious or noninfectious), or a history of chronic intraocular inflammation. Patients with any macular disease affecting vision were considered ineligible. Patients with a history of glaucoma or retinal detachment were also specifically excluded from the study, regardless of visual prognosis.

The primary outcome was the magnitude of net postoperative rotation of the toric IOL, measured at each scheduled postoperative study visit. Secondary endpoints of interest included the proportion of eyes with final net postoperative rotation of 5 degrees or less; the proportion of eyes with absolute residual astigmatism prediction error ≤ 0.5 D; the proportion of eyes with residual astigmatism ≤ 0.5 D and ≤ 1.00 D; and visual acuity outcomes, including monocular uncorrected distance (UCDVA) and intermediate (UCIVA), best spectacle-corrected distance (BSCDVA), and best distance spectacle-corrected intermediate (DSCIVA) visual acuities. Post hoc subgroup analysis was performed to investigate the effect of large (greater than 5 degree) intraoperative IOL rotations prompted by intraoperative aberrometry (IA).

### Preoperative workup

All patients underwent a complete ophthalmic history and exam, including subjective manifest refraction, intraocular pressure (IOP) measurement, slit lamp exam, and dilated fundoscopic exam. Digital alignment data using limbal registration were also captured preoperatively for all patients. Biometry was performed with a LENSTAR 900 (Haag-Streit USA, Mason, OH) optical biometer. Best corrected and uncorrected photopic visual acuity without glare, manifest refraction, intraocular pressure, slit lamp exam, dilated fundoscopic exam, and IOL orientation were obtained at all scheduled postoperative clinic visits; these were conducted approximately 1 day, 1–2 weeks, and 1 month after surgery (hereafter POD#1, POW#1, and POM#1). Postoperative IOL orientation was measured using digital photography with a slit lamp-mounted iPhone (Apple; Cupertino, California, USA), utilizing the toric reticle on the toriCAM app (Graham Barrett; version 4.0) as a reference mark. Photographs were then analyzed to determine the IOL axis. Patient medications, adverse events, device deficiencies, and subject-reported symptoms were documented at each visit during the postoperative period.

### Surgical technique

The Barrett Universal 2 toric formula was used for IOL power calculations and served as the basis for calculation of absolute residual astigmatism prediction error. A plano refractive target was chosen for all eyes. All surgery was performed by an experienced cataract surgeon (KB). An intraocular lens model number DFT315 or DAT315; DFT415 or DAT415; or DFT515 or DAT515, hereafter referred to as “T3,” “T4,” or “T5,” was implanted into the capsular bag using standard small-incision phacoemulsification techniques with a temporal clear corneal incision. No relaxing incisions were performed. The VERION™ digital marking system was used intraoperatively to guide and confirm alignment of the toric IOL. Intraoperative aberrometry (IA) using the ORA System® (Alcon) was also used to guide selection of toric IOL power and to verify optimal alignment of the IOL. If the IOL orientation was changed based on IA, the final orientation (“implantation axis”) was recorded with the VERION™ system and used as the baseline from which to assess postoperative rotation. Absolute postoperative rotation of an IOL at a particular point in time was defined as the absolute value of the difference between the implantation axis and the axis measured at the specified time point.

### Data analysis

Data were analyzed using Wizard 2.0.5 on OS X. All statistical tests were two-tailed, with a *P* value of 0.05 chosen as the definition of statistical significance. Data were approximately normally distributed except where noted. IOP data did not appear to be normally distributed and were evaluated using the Friedman non-parametric test for unequal ranks. Visual acuity and refractive outcome time series were characterized using a one-way *ANOVA* test. Data regarding the proportion of eyes achieving final refractive endpoints were evaluated with a chi-square test. Post hoc subgroup analysis was performed using a *t* test for equal means except as noted. Results are presented as value ± standard error unless otherwise specified. Visual acuity is presented in units of logMAR, except where otherwise specified.

## Results

### Demographics

A total of 35 eyes of 35 patients were recruited. Data for the POM#1 visit were unavailable for one patient. All other enrolled patients completed all study visits. Preoperative uncorrected visual acuity data were unavailable for two patients. Demographic data are reported in Table 1. There was a trend toward female predominance that did not reach statistical significance (*P* = 0.063). The axial length of all enrolled eyes fell within the range of 22–26 mm. A majority of eyes (74%) received a T3 lens, with smaller numbers of eyes receiving T4 (17%) and T5 (8.6%) lenses.

**Table 1:** Demographic data

Parameter		Minimum	Maximum	Mean (SD)	
Age (years)		54	82	68 (7.5)	
				Proportion (*n*)	
Sex	Male			34% (12)	*P* = .063*
	Female			66% (23)	
Operative eye	Right			46% (16)	*P* = .61
	Left			54% (19)	
Toric power	T3			74% (26)	
	T4			17% (6)	
	T5			8.6% (3)	
Total (*N*)				(35)	

*P* values were calculated using a one-proportion *z* test

T3, T4, and T5 refer to implantation of the DFT315 or DAT315; DFT415 or DAT415; and DFT515 or DAT515 intraocular lenses, respectively

### Safety

Median intraocular pressure (IOP) at baseline was 15. Statistically significant differences (*P* = 0.004) were found among the median postoperative IOP values: median IOP at POD#1 was slightly higher than baseline, by 1.5 mm Hg, but normalized on subsequent visits.

There were no major adverse events during the study. Any complications were minor and consistent in severity and frequency with those expected to occur with routine cataract surgery. No patient required return to the operating room for rotation of a toric IOL.

### Rotational stability

The mean absolute postoperative IOL rotation at POM#1 was 1.1 ± 0.2 degrees. This was stable throughout the postoperative period (*ANOVA*, *P* = 0.58) when measured at POD#1 and POW#1 (see Table 2). The maximum observed value of postoperative IOL rotation at POM#1 was 3.0 degrees. Therefore, one of the secondary endpoints of the study, the proportion of IOLs undergoing less than 5 degrees of net postoperative rotation, was met by 100% of eyes for which POM#1 data were available.

**Table 2:** Astigmatic refractive outcomes

Time Point	Absolute postoperative IOL rotation (degrees)	Residual refractive astigmatism (D)	Residual refractive astigmatism ≤ 0.5 D, % (*n*)	Residual refractive astigmatism ≤ 1.0 D,% (*n*)	Absolute residual astigmatism prediction error ≤ 0.5 D,% (*n*)	Total (*N*)
Predicted		0.23 ± 0.034^a^				35
POD#1	1.3 ± 0.28	0.19 ± 0.040	33 (94%)	35 (100%)	33 (94%)	35
POW#1	1.5 ± 0.26	0.20 ± 0.047	31 (89%)	35 (100%)	30 (86%)	35
POM#1	1.1 ± 0.22	0.21 ± 0.047	32 (94%)	34 (100%)	32 (94%)	34
*P* value	*P* = .58^b^	*P* = .91^b^	*P* = .58^c^	equal distribution	*P* = .34^c^	

*a*, *t* test for equal means between predicted and POM#1 residual astigmatism had *P* = 0.730; *b*, one-way *ANOVA* for equal means; *c*, chi-square test

POD#1, postoperative day 1; POW#1, postoperative week 1; POM#1, postoperative month 1

When eyes were grouped based on whether the amount of intraoperative IOL rotation performed as a result of guidance provided by IA was greater than, or less than or equal to, 5 degrees (see Table 3), there was no significant difference in mean absolute postoperative rotation between the two subgroups (1.57 ± 0.57 degrees vs. 0.96 ± 0.23 degrees, respectively; *P* = 0.26).

**Table 3:** Subgroup analysis of visual acuity and refractive outcomes at postoperative month 1

	Intraoperative aberrometry-guided IOL rotation		
Outcome	≤ 5 degrees (*n* = 27)	> 5 degrees (*n* = 7)	*P* value
BSCDVA	0.078 ± 0.020	0.081 ± 0.023	*P* = .95
UCDVA	0.17 ± 0.025	0.21 ± 0.047	*P* = .48
DSCIVA	0.17 ± 0.029	0.18 ± 0.059	*P* = .79
UCIVA	0.27 ± 0.041	0.28 ± 0.125	*P* = .89
Absolute residual astigmatism prediction error ≤ 0.5 D, *n* (%)	26 (96%)	6 (86%)	*P* = .28^a^
Residual refractive astigmatism (D)	0.20 ± 0.055	0.25 ± 0.094	*P* = .70
Absolute postoperative IOL rotation (degrees)	0.96 ± 0.23	1.57 ± 0.57	*P* = .26

Eyes were grouped based on whether the axis of the IOL was adjusted intraoperatively by 5 degrees or more as a result of intraoperative aberrometry guidance. Visual acuity is reported as logMAR ± SEM. *P* values were calculated using a *t* test for equal means, except as follows. *a*, *P* value was calculated using a two-proportion *z* test for equal means

BSCDVA, best spectacle-corrected distance visual acuity; UCDVA, uncorrected distance visual acuity; DSCIVA, best distance spectacle-corrected intermediate visual acuity; UCIVA, uncorrected intermediate visual acuity

### Visual acuity

Mean BSCDVA improved from 0.27 ± 0.030 (Snellen 20/37) preoperatively to 0.078 ± 0.017 (Snellen 20/24) at POM#1 (*ANOVA*, *P* < 0.001) (Table 4). Mean UCDVA improved from 0.93 ± 0.096 (Snellen 20/170) to 0.18 ± 0.022 (Snellen 20/30) at POM#1 (*ANOVA*, *P* < 0.001). Preoperative intermediate visual acuity was not assessed in this study; however, mean postoperative DSCIVA was 0.17 ± 0.025 (Snellen 20/30), and mean postoperative UCIVA was 0.27 ± 0.040 (Snellen 20/37).

**Table 4:** Monocular visual acuity outcomes

Time Point	BSCDVA (*N* = 35)	UCDVA (*N* = 35)	DSCIVA (*N* = 35)	UCIVA (*N* = 35)	Total (*N*)
Preoperative	0.27 ± 0.030	0.93 ± 0.096^a^	ND	ND	35
POD#1	0.42 ± 0.044	0.52 ± 0.047	0.70 ± 0.071	0.82 ± 0.068	35
POW#1	0.075 ± 0.013	0.18 ± 0.024	0.19 ± 0.028	0.27 ± 0.032	35
POM#1	0.078 ± 0.017	0.18 ± 0.022	0.17 ± 0.025	0.27 ± 0.040	34
*P* value	*P* < .001*	*P* < .001*	*P* < .001*	*P* < .001*	

Visual acuity is reported as logMAR ± SEM. *P* values were calculated using one-way *ANOVA*. *a*, preoperative uncorrected visual acuity data were unavailable from two patients, so *N* = 33 for this value

BSCDVA, best spectacle-corrected distance visual acuity; UCDVA, uncorrected distance visual acuity; DSCIVA, best distance spectacle-corrected intermediate visual acuity; UCIVA, uncorrected intermediate visual acuity; ND, not determined

Subgroup analysis based on the amount of IA-guided rotation found no statistically significant differences in any measured visual acuity outcome between the two subgroups. (see Table 3).

### Refractive outcomes

**Fig. 1:**
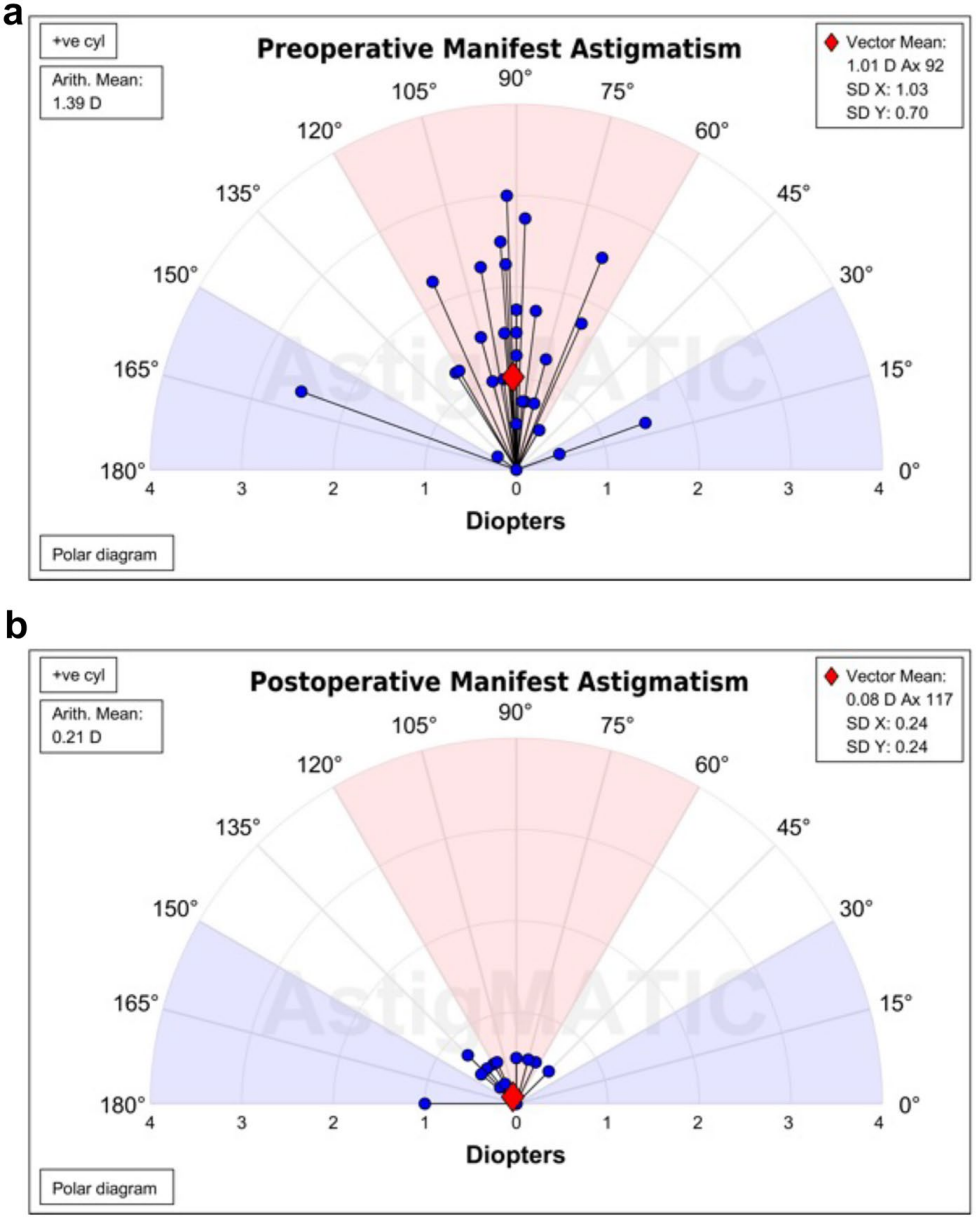
Preoperative and postoperative refractive regular astigmatism. The cylindrical component of preoperative and postoperative (month 1) manifest refractions were plotted [30] on a single-angle plot in positive cylinder notation

Mean residual regular astigmatic refractive error at POM#1 (Table 3) was 0.21 ± 0.047 D and was not statistically distinguishable at any time point (*ANOVA*, *P* = 0.91) from the mean residual astigmatism predicted during treatment planning. 94% of eyes achieved a final residual astigmatic refractive error of less than or equal to 0.5 D, and 100% had less than or equal to 1.0 D (Table 3). 94% of eyes were found to have a residual astigmatic refractive error at POM#1 that was within 0.5 D of the value predicted during treatment planning. There were no statistically significant differences in these proportions at any postoperative time point. Pre- and postoperative astigmatic refractive outcomes were visualized in a double-angle plot (Fig. 1).

When refractive outcome data were stratified on the amount of IA-guided rotation (Table 3), there were no statistically significant differences in residual refractive astigmatism (0.20 ± 0.055 vs. 0.25 ± 0.094; *P* = 0.70) or absolute residual astigmatism prediction error less than or equal to 0.5 D (96% vs. 86%; *P* = 0.28) between the low rotation (< 5 degree) and the high rotation (> 5 degree) subgroups, respectively.

## Discussion

To our knowledge, this is the first publication that reports real-world performance of the toric model of the DFT/DATx15 EDOF IOL.

A number of previous reports using physically similar, monofocal AcrySof® toric IOLs found mean absolute postoperative rotation of approximately 3.5–4 degrees [20–24, 26, 27], although a handful of studies have reported smaller values, such as 1.6 degrees [28] or 2.66 degrees [29], or reported a median (2 degrees [25]) rather than a mean. Our corresponding figure was 1.1 degrees, with a 95% CI of 0.7–1.5 degrees. Differences in inclusion criteria, patient population, surgical technique, and/or IOL orientation measurement technique, as discussed below, could account for the smaller mean postoperative rotations seen in this study compared to prior work. Our result supports the hypothesis that the rotational stability of the toric DFT/DATx15 is noninferior to that of other AcrySof® toric IOLs.

It is of clinical interest to compare this study's refractive outcome data to previous data [17–19] regarding the (non-toric) DFT/DAT015 EDOF IOL with the understanding that differences in inclusion criteria and methodology limit the validity of this comparison. The PMA study [17] and the more recent report by Bala et al [19] examined the DFT/DAT015 in patients without significant corneal astigmatism. Mean monocular BSCDVA in this study (0.078 ± 0.017; approx. Snellen 20/24) was slightly less than that reported for the PMA data (0.016 ± 0.0091; Snellen 20/21) and that of Bala et al [19] (–0.008 ± 0.0076; Snellen 20/20). Monocular UCDVA was not reported by either study. Monocular DSCIVA values were similar among the studies (this study, 0.169 ± 0.025; Bala et al., [19] 0.161 ± 0.0136; PMA data [17], 0.148 ± 0.0120). Small BSCDVA disparities could reflect characteristics of the different study populations. In addition to intrinsic differences in the amount of corneal astigmatism, the populations could also plausibly differ in the prevalence of comorbidities such as higher-order corneal aberrations. Inclusion and exclusion criteria were also dissimilar; for example, the PMA study excluded all “clinically significant ocular surface disease that would affect study measurements,” [17] whereas such disease was excluded in our study only if it limited visual prognosis. The time interval between our final visit, at 1 month, and the defined endpoints of the other two studies, at 6 months, could allow visual changes to occur due to postoperative evolution of the ocular surface or neuroadaptation. Other differences in data collection and reporting methodology, surgical planning and technique, and demographics, as well as any hypothetical differences attributable to use of the lens itself, could also explain this result. Additional studies would be needed to confirm the existence of this small numerical monocular BSCDVA difference, evaluate monocular UCDVA, and assess any clinical significance.

One strength of this study was the use of digital marking to maximize measurement accuracy of IOL orientation. There are several potential sources of error in the quantitative assessment of IOL rotational stability, and no gold standard methodology exists. The implantation axis can be defined by the intended placement axis or can be measured either intraoperatively or in the immediate postoperative period. Definition of implantation axis by postoperative measurement carries the risk that rotation that occurs between the time of implantation and the time of axis measurement will not be recorded; indeed, one study [31] found that rotation during the first postoperative hour constituted the largest component of the final net postoperative rotation. Preoperative definition and intraoperative measurement of the reference axis both rely on accurate marking of the orientation of the eye and measurement of the orientation of the IOL relative to the marks. Several studies (reviewed by Panagiotopoulou et al [32]) have found intraoperative digital marking systems, including the VERION™, to be equivalent or superior to manual marking.

Our study defined the implantation axis as that measured by the VERION™, thereby eliminating any effects attributable to inaccuracy of manual axis marking. Thus, the accuracy of the implantation axis measurement in our study was limited only by the accuracy of the VERION™ Digital Marker. In the absence of a gold standard methodology, the absolute accuracy of any given marking device is difficult to determine. One study [33] comparing two different intraoperative digital marking systems, including the VERION™, found alignment discrepancies between the two devices of 3 degrees or more in 47% of cases; however, absolute accuracy was not assessed, and it is not known which device, if either, was superior.

Postoperative measurement of IOL orientation can be accomplished with slit lamp techniques, optionally incorporating digital photography, digital image analysis [29], and/or use of custom software [28, 34]. In our study, postoperative IOL axis was measured by analyzing digital images taken from a slit lamp-mounted smartphone running the toriCAM app. In the setting of a dilated pupil, this software can acquire an image of the IOL orientation markings and overlay a toric reticle oriented by gravity. This procedure does not account for cyclorotation and is dependent on the accuracy of the accelerometer and camera hardware on the individual smartphone used for the measurement; no study in the open literature, to our knowledge, has assessed performance of the toriCAM app for postoperative measurement of IOL orientation. However, the accuracy of preoperative marking of the eye using the toriCAM app has been formally evaluated [35], using the iTrace wavefront aberrometer/topographer as a reference. Mean absolute error was found to be 1.28 ± 1.34 degrees, which could plausibly be interpreted as an upper bound on the error associated with reticle placement when using the toriCAM app.

For the purpose of toric IOL alignment, some recent studies have found advantages to the use of IA; however, not all studies concur, and the benefit may depend on the IOL formula against which it is compared [36–44] (reviewed in Kane [45]). Our methodology allowed us to capture all rotations prompted by IA. We defined a subgroup of eyes for which this rotation was more than 5 degrees and detected no outcome differences between this subgroup and the remainder of the study population. Because IA was utilized for all patients, and the sample size of the high rotation subgroup was particularly limited, the effect of IA cannot be reliably inferred from our data set alone. However, malrotation of a toric IOL by 5 degrees corresponds to a theoretical loss of approximately 17% of the astigmatic effect [46, 47]. This corresponds to roughly 0.25 D of lost astigmatism correction for a T3 lens, although in general the baseline residual astigmatism and lost astigmatism correction do not share a common axis and do not add linearly. Taking note of this estimate and the actual mean residual astigmatism of only 0.25 ± 0.094 D in the high rotation subgroup, it seems plausible to speculate that there could have been a clinically significant increase in residual astigmatism in this subgroup if IA had not been used. It therefore bears mentioning that the results of this study with regard to uncorrected visual acuity and astigmatic refractive outcomes may not be fully generalizable to settings in which IA is not routinely employed.

Limitations of this study include the following: in the absence of a control group, caution must be used when comparing our outcome data with results obtained using other lens options. The ability to detect rare adverse events and to perform subgroup analysis was limited by sample size. Patients with visually or surgically significant ocular comorbidities were excluded, so evaluation of the performance of the study IOL in such patients will require further investigation. Data regarding contrast sensitivity, mesopic visual acuity, and visual acuity in the presence of glare could also be collected in future studies. Subjective information regarding patient experience was not collected in a systematic manner, and the single-surgeon design and systematic use of IA and digital marking could limit generalizability with regard to refractive outcomes. Finally, although this study had no specific exclusion criteria based on biometric parameters other than corneal astigmatism, there were no eyes with extreme values of axial length enrolled in the study, which could also limit generalizability.

## Conclusion

The DFT/DATx15 toric EDOF IOL displayed excellent postoperative rotational stability, as anticipated given its strong physical similarity to other toric intraocular lenses from the same manufacturer. When used in combination with a digital marking system and IA, it yielded effective and predictable correction of astigmatism. No lens rotated postoperatively by more than 3 degrees at the final visit. Cautious comparison of visual acuity outcomes with outcomes reported in other studies for the non-toric DFT/DAT015 EDOF IOL noted similar monocular DSCIVA but a possible small disparity in monocular BSCDVA that is of uncertain origin and clinical significance and could readily be attributed to differences in patient population and/or study parameters. The toric DFT/DATx15 IOL had a good safety profile, consistent with previous data regarding the DFT/DAT015 IOL.

## Value statement

What was known.

- Toric intraolcular lenses (IOL) can help correct regular corneal astigmatism at the time of cataract surgery
- The DFT/DATx15 lens has been shown to yield excellent visual acuity at results in both distance and intermediate distance vision
- IOL rotation following IOL implantation has been documented to varying degrees

What this paper adds

- The DFT/DATx15 Toric IOL is rotationally stable
- The DFT/DATx15 Toric IOL effectively and predictably corrected regular corneal astigmatism
- The DFT/DATx15 Toric IOL yielded comparable uncorrected, intermediate visual acuities
